# *Echinococcus vogeli* in Immigrant from Suriname to the Netherlands

**DOI:** 10.3201/eid2103.141205

**Published:** 2015-03

**Authors:** Kees Stijnis, Anneke C. Dijkmans, Aldert Bart, Lodewijk A.A. Brosens, Birgit Muntau, Christoph Schoen, Thomas F. Barth, Thomas van Gulik, Tom van Gool, Martin P. Grobusch, Dennis Tappe

**Affiliations:** University of Amsterdam, Amsterdam, the Netherlands (K. Stijnis, A. Bart, L.A.A. Brosens, T. van Gulik, T. van Gool, M.P. Grobusch);; Medical Center Haaglanden, The Hague, the Netherlands (A.C. Dijkmans);; University Medical Center Utrecht, Utrecht, the Netherlands (L.A.A. Brosens);; Bernhard Nocht Institute, Hamburg, Germany (B. Muntau, D. Tappe);; University of Würzburg, Würzburg, Germany (C. Schoen);; University of Ulm, Ulm, Germany (T. F. Barth)

**Keywords:** *Echinococcus vogeli*, polycystic echinococcosis, immigrant, Suriname, PCR, immunohistochemistry, Em2G11, EM10, Netherlands, parasites

**To the Editor:** Neotropical echinococcosis, caused by polycystic larvae of the tapeworm *Echinococcus vogeli* and unicystic larvae of *E. oligarthrus*, is an emerging infection in rural South America (*1*,*2*). The parasites are propagated in a predator–prey cycle; the final and intermediate hosts for *E. vogeli* are bush dogs (*Speothos venaticus*) and pacas (*Cuniculus paca*), respectively (*1**,**2**)*. Human infections occur in rural areas and have been reported from several South American countries, mostly Brazil (*1*–*3*). Prompted by the recent diagnosis of an *E. vogeli* infection in a Surinamese patient in the Netherlands (*4*), we performed a retrospective analysis of all recent echinococcosis cases seen at the Amsterdam Medical Center. We describe molecular and immunohistochemical analyses from another case of *E. vogeli* infection.

In 2009, a 48-year-old female schoolteacher from Suriname sought care at the Amsterdam Medical Center for recently increasing retrosternal pain. Born in rural Suriname, she moved to the capital city of Paramaribo at 2 years of age. She had worked in the Brokopondo District for 1 year, then worked in urban Morocco, and immigrated to the Netherlands in 1990. Physical and laboratory examination findings were unremarkable. Esophago-gastro-duodenoscopy showed no abnormality. Abdominal ultrasonography and subsequent computed tomography revealed a lesion with solid and liquid components in liver segment 4, considered consistent with a biliary cystadenoma or an echinococcal cyst. Result of an echinococcosis indirect hemagglutination test with *E. granulosus* hydatid fluid antigen (Fumouze, Levallois-Perret, France) was strongly positive (titer 1:2,560; cutoff 1:160). An uncomplicated central liver resection of an 8-cm polycystic tumor was performed. Microscopic examination of resected tissue found vesicles containing protoscolices surrounded by periodic acid-Schiff–positive membranes. 

Based on these findings, the initial diagnosis was cystic echinococcosis caused by *E. granulosus*, most likely contracted in Morocco. Postoperative treatment was albendazole, 400 mg twice daily for 8 weeks. Findings from a 5-year follow-up examination were unremarkable.

Current histologic reanalysis from archived formalin-fixed paraffin-embedded surgical specimens revealed laminated layers of the parasites, characteristic of *E. multilocularis* and *E. granulosus* larvae (i.e., thin convoluted and very thick areas, respectively). All *Echinococcus* species can be distinguished by the size and form of their rostellar hooks from protoscolices (*1*); for protoscolices from the patient reported here, the mean lengths of the small and large hooks were 34 and 43 μm, respectively.

We performed PCRs of the cestode-specific 12S rRNA gene (*4*) and cytochrome oxidase subunit 1 (*cox1*) (*5*). BLAST (http://blast.ncbi.nlm.nih.gov) analysis of the 282-bp and 375-bp amplicons, respectively, showed 100% and 99% homology with *E. vogeli* (GenBank accession nos. KM588225, KM588226). Phylogenetic modeling based on the *cox1* sequence showed that this *E. vogeli* isolate clustered with isolates from Colombia and Brazil (Technical Appendix). Immunohistochemistry with monoclonal antibody Em2G11 raised against an *E. multilocularis* laminated layer antigen (Em2) (*6*) showed a faint and patchy pattern of the laminated layer in the *E. vogeli* lesion (Figure). Neither the previously described typical complete staining of the laminated layer as found in *E. multilocularis* larvae nor the entire absence of staining as described for *E. granulosus* metacestodes (*7*) was seen in the *E. vogeli* lesion. The typical staining of small particles of *E. multilocularis*, characteristically seen adjacent to *E. multilocularis* (spems) vesicles (*7*), was completely absent in this specimen. Immunohistochemical examination with a monoclonal antibody against echinococcal cytoskeleton protein EM10 (*8*) showed staining of the germinal layer and protoscolices of *E. multilocularis* and *E. granulosus* larvae but only partial staining of the protoscolices of *E. vogeli* larvae. According to the proposed staging scheme for polycystic echinococcosis (*1*), this case was assigned to stage 1.

**Figure:**
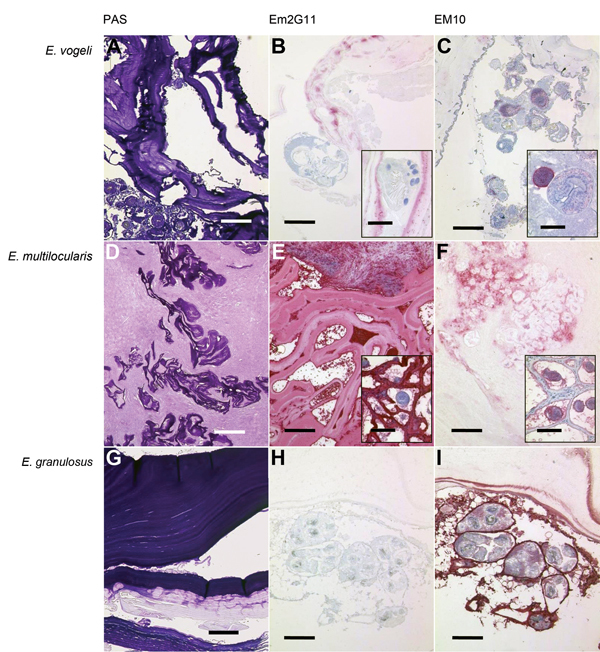
Periodic acid–Schiff (PAS) staining and immunohistochemical analysis of samples from *Echinococcus vogeli* lesions, with monoclonal antibodies against Em2 and EM10 of *E.* *vogeli* (A, B, C), *E. multilocularis* (D, E, F), and *E. granulosus* (G, H, I) lesions. Staining was performed on archived tissue from human patients with alveolar and cystic echinococcosis for comparison, and from the patient with *E. vogeli* infection who immigrated to the Netherlands from Suriname (*E. vogeli* infection in 2009). B and C insets) Protoscolex, with rostellar hooks clearly visible in inset B. E and F insets) Tissue from infected rodents (laboratory-infected *Meriones unguiculatus* gerbils) because *E. multilocularis* lesions in humans only rarely contain protoscolices. A, D, G) PAS-stained sections of the respective echinococcal lesions. Scale bars indicate 500 μm. B, E, H) Lesions with *E. vogeli*, *E. multilocularis*, and *E. granulosus* infection, respectively, stained with the monoclonal Em2G11 antibody against Em2 (for staining details see [*7*]). *E. multilocularis* lesions show intense staining, *E. granulosus* lesions show no staining, and *E. vogeli* lesions show patchy stains. Scale bars indicate 200 μm; scale bars of the insets indicate 50 μm. C, F, I) Respective lesions stained with antibodies against EM10 (dilution of the primary antibody 1:50; further steps as in Barth et al. [*7*]). Germinal layer and protoscolices of *E. multilocularis* and *E. granulosus* larvae are stained, but the protoscolices of the *E. vogeli* metacestode are only partly stained. Scale bars indicate 200 μm; scale bars of the insets indicate 50 μm.

Approximately 220 *E. vogeli* infections have been reported, including 10 from Suriname (*1*,*4*,*9*) and the case reported here. Only 1 case outside echinococcosis-endemic areas has been described in 2013; namely, in a patient from rural Suriname who immigrated to Amsterdam (*4*). The striking similarities between both cases extended to their clinical presentations.

In a recent immunohistochemistry study (*7*), antibodies against Em2G11 have shown excellent properties for distinguishing between cystic and alveolar echinococcosis. Although reported to not cross-react with purified laminated layer fractions from in vitro–kept *E. vogeli* (*10*), antibodies against Em2G11 exhibited an unusual and possibly discriminatory staining pattern when applied to the *E. vogeli* lesion from the patient reported here. Antibodies against EM10, which has not before been used for species discrimination on tissue sections, have also shown different staining properties.

Our findings suggest that there may be more undiagnosed cases of polycystic neotropical echinococcoses in immigrants from South America. In retrospect, the treatment (although aimed at *E. granulosus*) was successful despite the polycystic and proliferative nature of *E. vogeli* lesions, as indicated by an uneventful prolonged follow-up period for this patient with a well-circumscribed liver lesion. If neotropical echinococcosis had been considered before surgery (on the basis of radiologic features and the patient’s origin), the management would also have included a preoperative and prolonged course of albendazole therapy.

Technical Appendix Molecular phylogenetic analysis of *Echinococcus* sequences, including 2009 *E. vogeli* isolate from immigrant from Suriname to the Netherlands.
