# Supplementary material for: Echinococcus vogeli in Immigrant from Suriname to the Netherlands
Source: Emerg Infect Dis. 2015 Mar;21(3):528–30. doi: 10.3201/eid2103.141205 (PMC4344275; doi:10.3201/eid2103.141205)
Supplement: Technical Appendix — Molecular phylogenetic analysis of Echinococcus sequences, including 2009 E. vogeli isolate from immigrant from Suriname to the Netherlands. [file 14-1205-Techapp-s1.pdf]

Article DOI: http://dx.doi.org/10.3201/eid2103.141205

Echinococcus vogeli in Immigrant from Suriname to the Netherlands

Technical Appendix

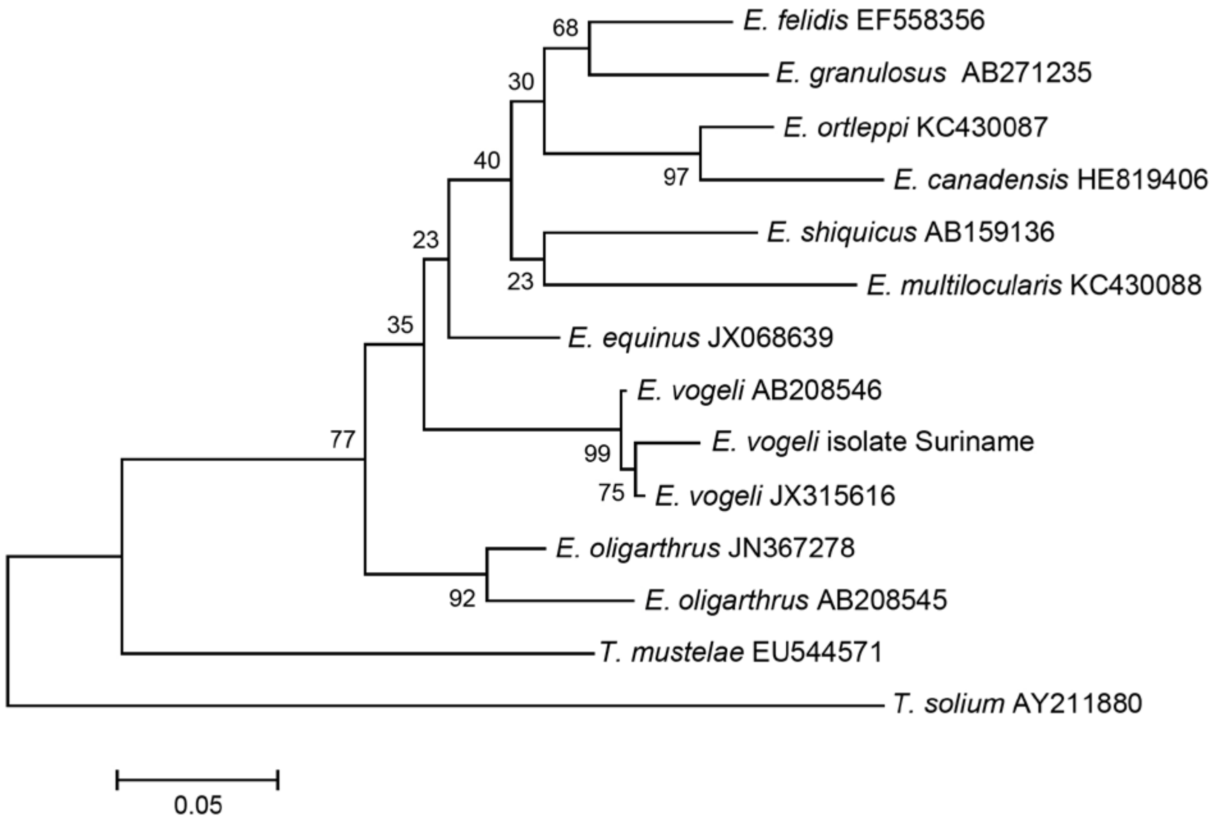

Technical Appendix Figure. Molecular phylogenetic analysis of *Echinococcus* sequences by the maximum-likelihood method. Numbers after the tapeworm species are GenBank accession numbers. The tree using cytochrome oxidase subunit 1 gene data depicts the isolate from immigrant from Suriname close to other *E. vogeli* specimens from Brazil (GenBank accession no. JX315616) and Colombia (AB208546). The evolutionary history was inferred by using the maximum-likelihood method based on the Tamura-Nei model. The tree with the highest log likelihood (–5295.5104) is shown. The percentage of trees in which the associated taxa clustered together is shown next to the branches. Initial tree(s) for the heuristic search were obtained automatically by applying neighbor-join and BioNJ algorithms to a matrix of pairwise distances estimated by using the maximum composite likelihood approach and then selecting the topology with superior log likelihood value. A discrete gamma distribution was used to model

evolutionary rate differences among sites (5 categories [+G, parameter = 0.2348]). The tree is drawn to scale; branch lengths are measured in the number of substitutions per site. The analysis involved 14-nt sequences. Codon positions included were 1st+2nd+3rd+noncoding. The final dataset contained a total of 1,752 positions. Evolutionary analyses were conducted by using MEGA6 (http://www.megasoftware.net).
